# Resilience of Coral-Associated Bacterial Communities Exposed to Fish Farm Effluent

**DOI:** 10.1371/journal.pone.0007319

**Published:** 2009-10-06

**Authors:** Melissa Garren, Laurie Raymundo, James Guest, C. Drew Harvell, Farooq Azam

**Affiliations:** 1 Marine Biology Research Division, Scripps Institution of Oceanography, University of California San Diego, La Jolla, California, United States of America; 2 Marine Laboratory, University of Guam, Mangilao, Guam; 3 Marine Biology Laboratory, Department of Biological Sciences, National University of Singapore, Singapore, Singapore; 4 Department of Ecology & Evolutionary Biology, Cornell University, Ithaca, New York, United States of America; University of Oxford, United Kingdom

## Abstract

**Background:**

The coral holobiont includes the coral animal, algal symbionts, and associated microbial community. These microbes help maintain the holobiont homeostasis; thus, sustaining robust mutualistic microbial communities is a fundamental part of long-term coral reef survival. Coastal pollution is one major threat to reefs, and intensive fish farming is a rapidly growing source of this pollution.

**Methodology & Principal Findings:**

We investigated the susceptibility and resilience of the bacterial communities associated with a common reef-building coral, *Porites cylindrica*, to coastal pollution by performing a clonally replicated transplantation experiment in Bolinao, Philippines adjacent to intensive fish farming. Ten fragments from each of four colonies (total of 40 fragments) were followed for 22 days across five sites: a well-flushed reference site (the original fragment source); two sites with low exposure to milkfish (*Chanos chanos*) aquaculture effluent; and two sites with high exposure. Elevated levels of dissolved organic carbon (DOC), chlorophyll *a*, total heterotrophic and autotrophic bacteria abundance, virus like particle (VLP) abundances, and culturable *Vibrio* abundance characterized the high effluent sites. Based on 16S rRNA clone libraries and denaturing gradient gel electrophoresis (DGGE) analysis, we observed rapid, dramatic changes in the coral-associated bacterial communities within five days of high effluent exposure. The community composition on fragments at these high effluent sites shifted towards known human and coral pathogens (i.e. *Arcobacter*, *Fusobacterium*, and *Desulfovibrio*) without the host corals showing signs of disease. The communities shifted back towards their original composition by day 22 without reduction in effluent levels.

**Significance:**

This study reveals fish farms as a likely source of pathogens with the potential to proliferate on corals and an unexpected short-term resilience of coral-associated bacterial communities to eutrophication pressure. These data highlight a need for improved aquaculture practices that can achieve both sustainable industry goals and long-term coral reef survival.

## Introduction

Reef-building corals are just one of many animals that have a mutualistic microbial community. However, the particular relationship between corals and their associated microbes may have a more direct linkage to ecosystem health compared with other species' symbiotic microbial community. The ecosystem-level influence of coral-associated microbial communities is rooted in the fundamental role that scleractinian (stony, reef-building) corals play in physically structuring the habitat and supporting reef organisms, and the roles coral-associated microbes have in maintaining holobiont health. In recent years, molecular methods have greatly expanded our ability to study coral-associated microbial communities. We now know some coral species-specific associations of microbes exist [1], that coral-associated communities are distinct from water column-associated microbes [1]–[3], and that the bacterial communities of corals can shift under conditions of stress or disease [4]–[7]. The causative agents of many coral diseases remain unknown [8], [9], as do the outcomes of interactions among coral-associated microbial communities and various environmental perturbations, such as nutrient enrichment.

Coral disease [10] and nutrient enrichment of coastal waters [11] actively contribute to the continued decline of coral reefs globally. As coastal development and coral disease incidence continue to rise [12], understanding the mechanisms linking them [13] and managing reefs for long-term survival become pressing management priorities.

Managing for resilience is a current priority in the field of coral reef management [14], [15]. The goal is to develop and employ management strategies that increase the ability of reef ecosystems to withstand and recover from stress. Three cornerstones have been proposed to aid the empirical assessment of ‘resilience’: biodiversity, spatial heterogeneity, and connectivity [15]. The focus has been on broad reef-wide or region-wide assessments [16], but a potentially important and overlooked component to consider is the resilience capability of the individual corals that comprise the reefs. At this scale, we must consider coral-associated microbial communities [4], [17]. The same three cornerstones of resilience can also be applied to the microbial communities within a single coral colony. Here we define resilience as the ability of the microbial communities to return to their original composition after disturbance by nutrient enriched waters despite the continued presence of this enrichment. The large body of work illuminating the role of coral-algal symbioses in large-scale bleaching patterns [18], [19] is a good example of how understanding the biodiversity, spatial heterogeneity, and connectivity of single-celled dinoflagellates (zooxanthellae) provided critical links between the small-scale processes of coral-algal associations and region-wide bleaching patterns. The discovery of a vast genetic diversity of zooxanthellae led to the realization that not all algal symbionts of corals are equally tolerant to environmental stressors. The distribution of algal symbionts that were more susceptible to increased temperature stress explained not only the single colony-scale bleaching patterns observed in some corals [18], but also helped explain larger region-wide bleaching patterns. It has become clear that the zooxanthellae diversity and distribution patterns of a reef are worth considering when planning management strategies for a given region [20]. The parallel body of work for coral-associated bacteria, archaea, and viruses is much younger and smaller than that of zooxanthellae. We currently understand more about the biodiversity of the coral-associated microbes than we do about the spatial heterogeneity or connectivity of these communities [21]. Thinking about the microbial ecology of these microscale ecosystems in the context of the three resilience criteria will help illuminate the functional role these organisms play in coral health and disease.

This study builds on previous research that demonstrated that coral reef water-associated microbial communities were influenced by effluent from coastal milkfish (*Chanos chanos*) aquaculture in Bolinao, Philippines [17]. Since the corals that lived in the channel before the intensive aquaculture industry began have died out (E. Gomez, pers. comm.), we simulated the effect of the introduction of these fish pens on corals by transplanting live, clonally replicated corals from a relatively effluent-naïve site (Reference site) to sites routinely exposed to some amount of effluent (low exposure as sites Far-1 & 2; high exposure at sites Near-1 & 2). We investigated the biodiversity of coral-associated bacteria communities over 22 days in response to this chronic effluent exposure. This allowed us to investigate both the effect of spatial proximity to fish pens and temporal transitions in microbial communities. We tested the hypotheses that: 1) exposure to fish pen effluent would cause shifts in coral-associated bacterial community composition towards phylotypes not normally associated with healthy corals; 2) corals exposed to high levels of effluent would be colonized by fish pen-associated bacteria; and 3) there would be some degree of resilience in bacterial communities allowing corals to maintain or recover their original microbial communities despite the stress of effluent exposure. This study broadens our knowledge of the links among environmental conditions, bacterial community composition, and coral health. In the face of rising coastal development and changing environmental stimuli, understanding these connections will aid in developing effective and ecologically sound coral reef management strategies. A focus on microbial community shifts in response to organically enriched effluent will contribute to understanding the connectivity of these communities on coral reefs, further advancing our understanding of how to manage for increased resilience.

## Results

### Fish Pen Effluent is Persistent, Spatially Extensive and Traceable in Real Time

**Figure 1:**
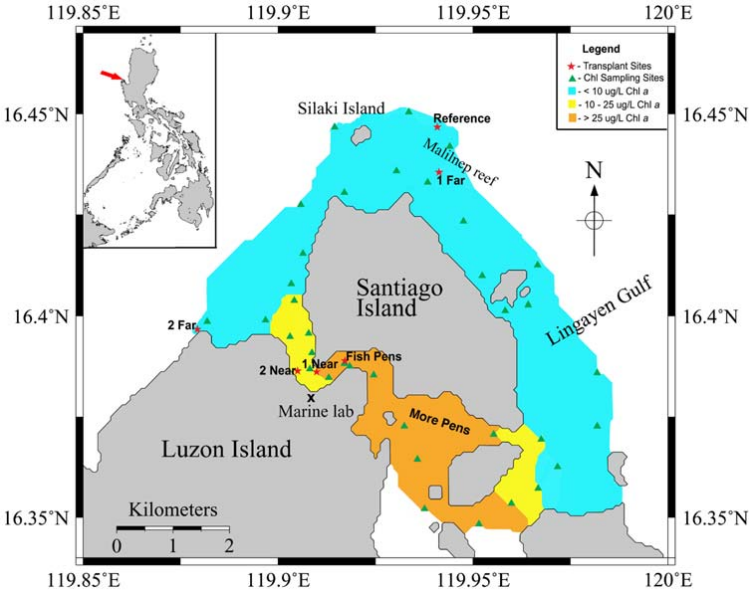
A map of the study sites and surface water *in vivo* chlorophyll *a* measurements averaged over 4 consecutive weekly samplings. Note that transplant site “Fish Pens” did not have corals placed there since no live coral currently exists at that site. It was the location of all water and sediment sampling for the experiment as a representative fish pen.

Weekly mapping at 20 stations all around Santiago Island showed that the strongest chlorophyll *a* signals consistently occurred in the channel containing the majority of fish pens (Fig. 1). There are no major river discharges or other point-sources of nutrient enrichment at or up-current (south east) from the fish pens [22], thus the majority of organic matter input can be attributed to the fish pens. An elongated plume of chlorophyll *a* in the direction of the prevailing current was also consistent with the hypothesis that the fish pens were the source of enrichment in this system.

Bacterial abundance, frequency of dividing cells (FDC), cyanobacteria abundance, virus-like particle (VLP) abundance, concentrations of colony forming units (CFU) of *Vibrio* and kanamycin-resistant bacteria, dissolved organic carbon (DOC) concentration, and chlorophyll *a* concentrations at sites along a strong fish pen gradient were all higher during sampling in May and June 2008 than a previous sampling period in January 2007 ([17],Table 1). A pronounced wet season (May and June 2008) influenced the gradient of fish pen effluent distribution. These data also suggested that corals at the most well-flushed sites (such as the Reference site; Fig. 1) in our study area were no longer living in an oligotrophic system. The DOC, bacteria, VLP, and chlorophyll *a* concentrations were all indicative of a mesotrophic or eutrophic ecosystem. The relatively high percentage of total cells that were dividing at any given time point at any site (ranging from 8.6±3.1% at Far-2 site to 17.0±1.1% at the Fish Pens) indicated that the enriched DOC observed at all sites (ranging from 77.5±0.8 µM at Far-1 to 189.8±1.0 µM at Fish Pens) was readily utilizable to the microbial community to support growth. A truly oligotrophic environment normally exhibits DOC concentration of ∼40 uM [23], which is much lower than our relatively well-flushed sites.

**Table 1:** Comparison of Water Characteristics.

Site	Average values from May and June 2008 (T0, T-5days, T-22days)								Values from January 2007 (Garren et al. 2008)			
	Free-living Bacteria (cells x 10^6^/ml±SE)	Virus-like Particles (VLP x 10^6^/ml±SE)	Extracted Chl *a* (µg/L±SE)	Dissolved Organic Carbon (µM±SE)	Frequency of Dividing Cells (% total abundance dividing±SE)	Cyanobacteria Abundance (cells x 10^4^/ml±SE)	Vibrio (TCBS media) plate counts (CFU/ml±SE)*	Kanamycin resistant plate counts (25 µg/ml; CFU/ml±SE)*	Free-living Bacteria Abundance (cells x 10^6^/ml±SE)	Virus-like Particles (VLP x 10^6^/ml±SE)	Extracted Chl *a* (µg/L±SE)	Dissolved Organic Carbon (µM±SE)
**Ref**	1.51±0.03	8.0±0.8	2.8±1.2	93.4±17.2	11.1±2.9	11.3±1.0	60±0	398±43				
**Far-1**	1.94±0.05	14.2±0.9	3.7±0.1	77.5±0.9	11.9±2.0	7.5±0.8	40±12	1765±1495	0.54±0.03	10±0.7	0.25±0.03	69.7±1.3
**Far-2**	1.76±0.04	14.8±1.0	4.1±1.6	82.0±4.6	10.5±2.8	9.9±1.2	53±7	285±95				
**Near-1**	7.43±0.18	81.4±4.3	63.4±12.9	143.6±1.4	12.8±0.8	63.4±4.6	160±23	1008±93	0.61±0.06	70±3	4.5±0.2	141±2.9
**Near-2**	5.71±0.15	75.9±3.6	60.2±15.3	158.0±2.5	12.4±1.9	52.2±5.9	220±23	1218±238				
**Pens**	10.25±0.50	112.3±4.7	99.8±25.2	189.8±1.0	16.7±1.4	64.2±8.0	340±46	1413±73	0.99±0.03	61±7	10.3±0.2	162±18.5

Water Characteristics from May/June 2008 compared to January 2007. (* signifies T0 data only).

### Coral-Associated Bacteria Communities Shift in Response to Effluent

**Figure 2:**
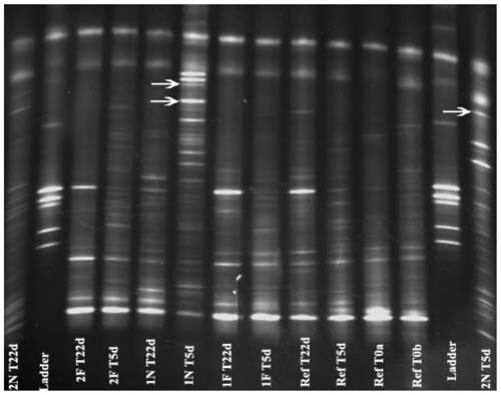
Denaturing gradient gel electrophoresis (DGGE) image of one coral colony (#1) at all sites (Far-1&2, Near-1&2, and Reference) across all time points (T-0, T-5 days, T-22 days). Arrows indicate *Desulfovibrio* bands that were sequence verified. There were two separate fragments sampled from each colony at T0. Both samples are shown on this gel as T0 a and b. Samples from the high effluent sites (Near-1&2) at T-5 days were the only visible *Desulfovibrio* bands.

One fragment out of the 40 transplants died at site Near-2 before the 5-day sampling point (T-5 days). All other fragments appeared visually healthy throughout the duration of the experiment. Large community shifts in the coral-associated bacteria samples were observed via denaturing gradient gel electrophoresis (DGGE) analysis five days after transplantation. Profiles were most radically different at the Near sites. However, changes were observed at all sites including the Reference, indicating a transplantation effect in addition to an effluent effect. By 22 days post-transplantation (T-22 days), all coral-associated microbial community profiles had shifted back towards their original profile patterns observed at T-0 (Fig. 2).

All corals transplanted to the Near sites at T-5 days showed, via DGGE profiles, a prominent band that matched the 16S rRNA sequence of the coral black band disease (BBD)-associated [24] bacterium *Desulfovibrio* sp. ([15]; Accession No. AY750147.1) with 100% similarity and 100% query coverage. The majority of the bands for this phylotype were present in coral fragments transplanted at sites Near-1 and Near-2 at T-5 days. This genus was not detected with genus-specific polymerase chain reaction (PCR) primers in the bacterial communities associated with the water column at any site and was only detectable in sediments from sites Near-2 and Fish Pens. Sequences related to the genus were not present in clone libraries for water or sediment bacterial communities. No coral fragment had detectable levels at T-0, but at T-5 days all corals had detectable levels at most sites, identified by genus-specific PCR analysis (Table 2). By T-22 days, only fragments at the Near sites had detectable levels of genus-specific PCR product remaining, the bands in DGGE were greatly diminished or absent, and no sequences were present in clone libraries from that time point.

**Table 2:** *Desulfovibrio* Presence/Absence.

Coral & Time	Colony 1		Colony 2		Colony 3		Colony 4	
Site	T-5d	T-22d	T-5d	T-22d	T-5d	T-22d	T-5d	T-22d
**Ref**	**+**	−	−	−	**+**	−	**+**	−
**Far-1**	−	−	**+**	−	**+**	−	−	−
**Far-2**	**+**	−	**+**	−	**+**	−	**+**	−
**Near-1**	**+**	**+**	**+**	**+**	**+**	**+**	**+**	**+**
**Near-2**	**+**	**+**	**+**	died	**+**	**+**	**+**	−

The presence (+) or absence (−) of *Desulfovibrio* as detected by genus-specific PCR amplification. No amplified product was detected in any colony at T0, so this time point is omitted from the table for simplicity. The T-22 days fragment for colony 2 died before T-5 days, and was the only mortality for the experiment. *Desulfovibrio* returned to undetectable levels for all colonies at all low-effluent sites by T-22 days (Ref, Far-1&2), yet remained detectable in most colonies at the high-effluent sites (Near-1&2).

**Figure 3:**
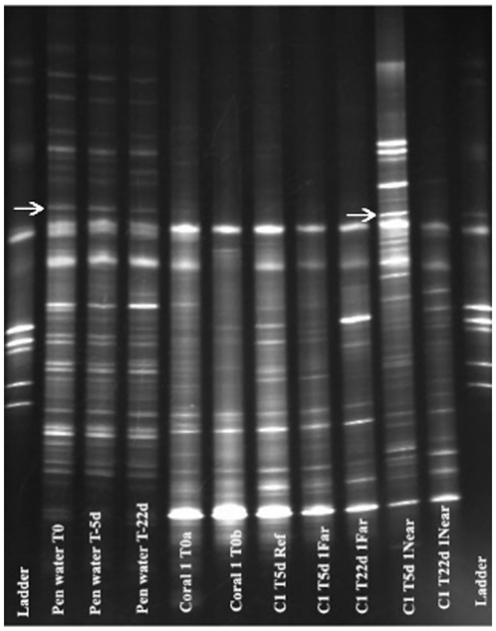
DGGE image showing *Roseobacter* band (>97% sequence similarity) at all time points in the Fish Pen water, but only T-5 days at site Near-1 for coral 1.

Evidence suggested that water-associated bacteria from the Fish Pens had the ability to colonize corals exposed to effluent-rich waters. A phylotype most closely related to an uncultured *Roseobacter* (Accession No. EU627982.1) comprised enough of the water-associated bacteria community at the Fish Pens to be detectable via DGGE at T-0, but was completely absent in coral-associated bacteria communities. However, five days after transplantation, this band (verified through sequence analysis) became strongly visible in corals transplanted to Near sites but not in the fragments of the same colony that were transplanted to Far sites (Fig. 3). This same sequence was observed in January 2007 clone libraries from free-living bacteria of fish pen water [17], suggesting that this bacterium may be a persistent feature of fish-pen influenced seawater in Bolinao.

**Figure 4:**
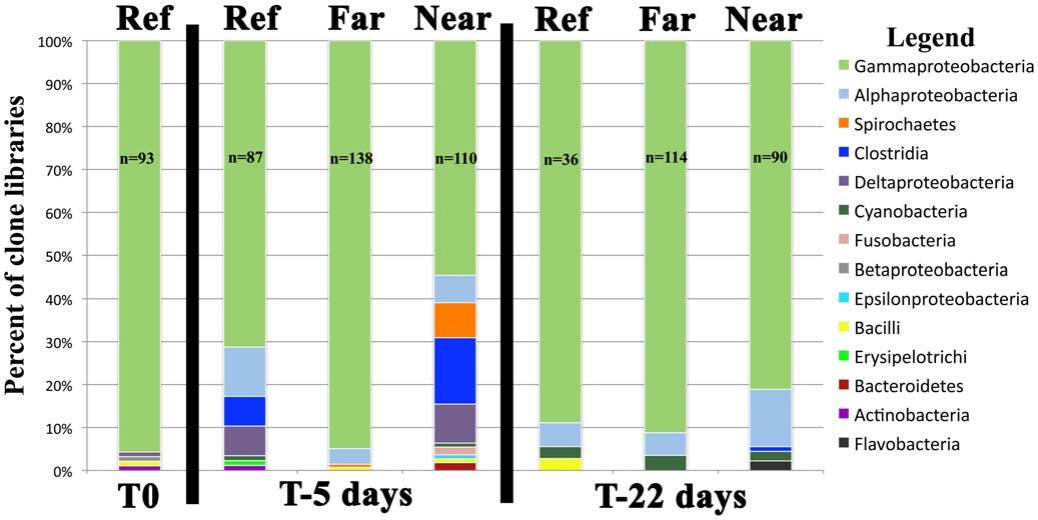
The distribution of phylotypes by class from 16S rRNA clone libraries. Libraries have been pooled by site (i.e. libraries from all fragments at a given time point from Far-1 and Far-2 are represented by “Far” and the same is true for “Near”) and each individual time point (T0, T-5 days, T-22 days) is shown separately. The number of sequences represented is denoted as “n = ”. Spirochaetes, previously seen only in corals infected with diseases, and Clostridia are both present even though fragments showed no visible signs of disease. Spirochaetes sequences are only present at T-5 days and predominantly at high effluent sites.

Clone libraries of coral fragments, water, and sediment revealed similar patterns to those seen via DGGE analysis. Very little overlap of phylotypes was observed among water, coral, and sediment bacteria communities. Of the 1172 sequences analyzed from all libraries, there were several noteworthy patterns. Eighteen sequences from the family Vibrionaceae were observed—14 of which came from corals at various site only during the T-5 days time point, while the rest were observed in sediments from the Near sites. Nineteen sequences from the genus *Desulfovibrio* were observed in coral clone libraries– all but two occurrences were from T-5 days and 11 were from fragments at Near sites. Thirty sequences from an order of anaerobes, Clostridiales, were also present among the coral libraries—all but one occurrence were from T-5 days and all but six were from Near sites. Only one Clostridiales sequence was observed in a non-coral library: Far-1 sediment at T-0. Four occurrences of the anaerobic sulfite-oxidizing genus *Sulfitobacter* were observed in a coral at the Reference site (n = 1) at T-5 days, and at Near-1 (n = 3) at T-22 days. Two genera were coral-specific, appearing in coral libraries from all time points at all sites: *Alcanivorax* (n = 18) and *Halomonas* (n = 334). Two instances of sequences belonging to genera predominantly comprised of pathogens (human, porcine, bovine, feline, canine, and equine) occurred on corals at the Near sites five days post-transplantation. *Arcobacter*, which is generally associated with feces (human, porcine, and bovine) and has been found in sewage-contaminated waters, was present on a coral at site Near-2 while *Fusobacterium*, often associated with necrotic lesions and a variety of mammal diseases, was present on a coral at site Near-1. Sequences from the class Spirochaete, which have been found previously in diseased coral samples in association with BBD and White Plague-like syndromes [2], [4], [25], occurred only at T-5 days, mostly at Near sites (Fig. 4).

RDP-10 LibCompare analysis indicated that T-5 days libraries for fragments from different coral colonies at Near sites were more similar to each other than they were to their own original colonies at T-0 or T-22 days (p≪0.05). In particular, the abundances of Bacteriodetes, Deltaproteobacteria, and Firmicutes were higher at T-5 days than any other time point (Fig. 4). Within a colony, the phylotype distributions were fairly similar among Reference, Far-1 and Far-2 for all time points and Near-1 & 2 for T-0 and T-22 days. The other clone library pattern that revealed itself was an apparent increase in diversity at the high effluent sites at T-5 days. The number of classes represented at the Near sites increased at T-5 days, while the number decreased at the Reference and Far sites (Fig. 4). The Shannon-Wiener index of diversity also increased by 1.7954, from 1.5358 at T-0 to an average of 3.3312 at T-5 days, at the high effluent sites. In contrast, the values for corals at the low effluent sites increased by an average of 0.5140, and by 0.3636 at the reference site.

## Discussion

There are several lines of evidence supporting our hypothesis that fish pen effluent induces changes in coral-associated bacterial community composition. Both DGGE and clone library community composition profiles from all coral colonies at T-5 days at high effluent (Near) sites were more similar to other colonies transplanted to those sites than they were to the communities associated with the fragments from their own colonies transplanted to lower effluent (Far or Reference) sites or sampled from the high effluent sites at the later time point (T-22 days). Corals at high effluent sites were also the only samples with detectable levels of *Desulfovibrio* genus-specific PCR products at the final time point, by which time the same coral clones at lower effluent sites had recovered their original bacterial community composition. This suggests that high effluent exposure was either a source or a facilitator for change in the coral-associated bacterial communities.

The ability of a fish pen-associated *Roseobacter* to become a prominent member of the DGGE profiles from corals at high effluent sites during the first few days post-transplantation was consistent with our hypothesis that corals could become colonized by aquaculture-associated bacteria. Sequences typing pathogenic genera (*Arcobacter* and *Fusobacterium*) generally associated with humans that became associated with the corals were only present at high effluent sites. The fish pens are tended by people who stay in structures directly above the pens. These facilities lack any form of septic or plumbing systems. The presence of potential human pathogens associating with corals suggests that these fish pens represent more than just a source of fish-associated microbes onto the reefs, and that corals are sensitive to these inputs. Beyond the implication for corals, the human health implications of these pathogenic bacteria should not be ignored. These waters are also used for swimming and recreation, thus current aquaculture practices may require improvement for both human and coral health.

A noteworthy observation during this experiment was the restoration of the microbial communities by day 22 towards their original T-0 composition across all sites, despite the surrounding water remaining essentially unchanged. Our original hypothesis was that the low effluent site corals would have the ability to maintain or restore their bacteria community composition, but we observed a much better recovery than anticipated in the high effluent site corals as well. This observation suggests that there is a preferred/stable coral-associated composition that the bacterial community tends toward, and generates hypotheses regarding mechanisms by which a conserved microbial community can be maintained. Whether the coral itself [26]–[29], its microbial associates [30], [31], some combination of the two, or another component or set of components of the holobiont is responsible for restoring the bacterial community balance remains to be understood. However, the observation of the community changing in response to effluent exposure and subsequently recovering *without* effluent reduction suggests that the mechanisms for bacteria community control are strong. Illuminating these mechanisms will be a critical step towards understanding the role of bacterial communities in coral health and disease, and facilitating predictions of reef-wide responses to various stressors.

The fact that the coral-associated bacterial communities at all sites, including the Reference, changed to some degree at T-5 days demonstrated that there was an effect of transplantation. The appearance of *Desulfovibrio* bacteria, generally associated with coral black band disease (BBD), on all coral samples and at all sites at T-5 days was in agreement with our hypothesis that stress, in this case induced by transplantation, would allow corals to be opportunistically colonized by potentially harmful bacteria. Due to the geography and hydrography of the area, the two Near sites had to be located closer to each other than the two Far sites. Thus, one might expect the Near sites to be more similar to each other than the Far sites would be. However, the RDP LibCompare results analyzing clone libraries showed that microbial communities from all fragments at both Near sites at T-22 days were more similar to the communities associated with any fragment from the Far or Reference sites (from any time point) than they were to the communities associated with fragments from the very same colony sampled at T-5 days at that same Near site.

Unlike the *Roseobacter* that was transferred from the fish pen effluent to the coral, the source of *Desulfovibrio* bacteria seems more likely to be the coral itself. Given that *Desulfovibrio* phylotypes were not detectable in water or sediment samples from most sites, yet were detectable in corals at all sites during at least one time point—as it has been detected in corals from around the globe [2], [32]— we hypothesize that this potentially detrimental phylotype was originally associated with the corals in undetectable numbers rather than with the surrounding environment. It seems likely that the stress of transplantation, through an unidentified mechanism, allowed these bacteria to proliferate rapidly. However, while transplantation likely initiated the proliferation, exposure to fish pen effluent seems the most likely cause for the continued proliferation of *Desulfovibrio*, as they were visible only at high effluent sites by the final time point. The same mechanisms controlling *Desulfovibrio* proliferation may also be responsible for the proliferation of Clostridia and Spirochaetes in this experiment. The two separate likely sources of newly colonizing bacteria, aquaculture effluent and the coral itself, highlights the potential for multiple pathways of microbial colonization to occur simultaneously. Investigating the ecological interactions among the coral animal, its associated microbiota, and water column-derived microbial invaders may shed light on synergistic roles played by host immunity [26]–[29], [33] and microbe-microbe interactions [9], [21], [30], [31] to protect the holobiont from undesired microbial colonization.

Though all but one of the 40 transplanted coral fragments survived the experiment, it is a worthwhile exercise to consider other potential trajectories this experiment could have taken and the possibilities for these fragments after the final time point. It seems that many of them, especially at the Near sites, may have been particularly vulnerable 5 days after transplantation given the relative increase of sequences affiliated with potential pathogens in molecular profiling results. A previous study showed decreased survival of juvenile corals at this site, and it is possible our corals could have followed a similar fate [22]. Had there been any additional stressor (such as increased water temperature or a major storm event), it is possible that we would have observed a drastically different survival rate. Also, the short time scale of this experiment cannot be extrapolated to imply that these fragments would have survived a long-term transplantation to these sites. The return of the bacteria communities towards their original structure seems to be a positive indication of more resilient capabilities than previously observed; however, the fact that some of the 5 day time point bacteria (such as members of *Desulfovibirio* and Clostridiales) were still detectable at the end of the experiment in corals at high effluent sites should not be overlooked. It is certainly possible that the persistence of these phylotypes could be an early indicator of future disease [34]. Also, the potential physiological costs –such as reduced growth, unsuccessful reproduction, or impaired ability to heal from wounds— of returning the bacteria communities to their original state after disturbance should be considered. There may be hidden costs of this response that we were unable to measure in this experiment.

Observing dramatic changes in community composition over the short time scale of 5 days calls attention to the sensitivity and susceptibility of these corals to physical stress and organic matter enrichment. The lack of visible signs of disease during this stress event raises the need to monitor and observe corals at smaller scales than current monitoring techniques allow. For instance, it may be informative to incorporate microscopy techniques into routine coral reef monitoring protocols. Studying the microbial ecology of sub-lethal and sub-visible effects of stress may provide some of the mechanistic links we need to understand and predict physiological responses of corals to various scenarios. These data provide insight into some of the microbial biodiversity that may be integral in resilience for these corals, and raise the question of whether or not increased diversity of associated bacteria is a desirable state for corals. Our data suggest that an increase in diversity could be indicative of, or correlated to, stress events. These data provide a broader understanding of potentially desirable and undesirable groups of bacteria through time, but they do not yet provide insight into the spatial heterogeneity at the colony scale or connectivity of these microbial consortia. To gain insight into those two resilience criteria, we must shift towards incorporating and developing more sophisticated visual methods of analysis. Some methods that could be incorporated in coral microbiological studies more routinely to this end include fluorescence *in situ* hybridization (FISH), confocal microscopy, and atomic force microscopy. Developing the proper tools to monitor these microscale microbial interactions *in situ* will increase our ability to understand and manage coral reefs in the face of a rapidly changing environment. On a positive note, the ability of the coral-associated bacterial communities to rebound and recover after this severe stress event, without any improvement in the water quality, suggests that corals may be able to survive short, severe stress events if they are not pushed beyond a threshold. The more we learn about individual coral resilience capabilities, the better we can develop management practices integrated across multiple spatial scales for the long-term success of reefs.

## Methods

### Study Sites

The study took place in the Bolinao, Pangasinan province, Philippines (16N, 119E). Milkfish (*Chanos chanos*) mariculture has been actively practiced in the area since 1995 [35]. The farms employ net pens measuring roughly 10 m×10 m×8 m with a stocking density of approximately 50,000 fish per pen and a pen density of 10 per hectare [22].

A site within the channel between Luzon and Santiago Island was chosen as a representative fish pen sampling site (referred to as “Fish Pens”) for water and sediment collection. Two reef sites <1 km from the Fish Pens were selected as sites Near-1 and Near-2 (high effluent exposure) and two reef sites >5 km from the Fish Pens were selected as sites Far-1 and Far-2 (low effluent exposure). The Reference site was ∼10 km from the Fish Pens on the outside of the Malilnep reef crest that is regularly flushed by the South China Sea (Fig. 1).

### Experimental Design

Four colonies of *Porites cylindrica* were selected from the Reference Site. Twelve branches from each colony were gently removed using wire cutters. Two fragments from each colony were immediately flash frozen as the T-0 sampling. Two additional fragments were transported and affixed in place at each of the following transplantation sites: Reference, Far-1, Near-1, Far-2, Near-2 (note: corals were not transplanted directly at Fish Pens because live coral does not currently exist in that location). The fragments were affixed at each site in flexible plastic tubing with their source colony labeled with Dymo tape and zip-tied to plastic mesh tables installed 1 m above the substrate on rebar supports (Fig. S1). Three replicate tables were installed at each site at 2–3 m depth with the fragments from each colony randomized among the three tables. One fragment from each colony was collected from each site 5 days after transplantation and the second fragment was collected 22 days after transplantation. All fragments were monitored at each visit for visible signs of stress or disease. The experiment took place between May 19 and June 10, 2008.

### Sample Collection

Four liter water samples were collected directly above the transplant tables at each site during each sampling period. They were kept cool and shaded until they were processed in the lab within 4 hr of collection. Triplicate sediment cores were collected with sterile 10 ml syringes from each site at T-5 days. Coral fragments were collected in sterile WhirlPaks (Nasco, USA), rinsed with sterile seawater, wrapped in aluminum foil, and flash frozen in liquid nitrogen.

### Sample preservation for DNA

A particle fraction was operationally defined as 3 µm being the minimum particle size [36], [37]. To preserve water samples for DNA extraction, 200 ml seawater were pre-filtered through a 3 µm-pore size filter (47 mm diameter, polycarbonate, Whatman) and the filtrate was put onto a 0.22 µm-pore size filter (47 mm diameter; Supor 200; Pall Corp.). The 0.22 µm filters were stored at −20°C in 250 µl of RNALater™ (Ambion, USA). To preserve coral samples for DNA extraction, frozen coral fragments were thawed on ice, airbrushed in 2 ml sterile seawater to remove tissue and mucus along the length of the fragment except from the portion that was in contact with the mounting tube, and stored at −20°C. Sediment cores were kept cold until they could be flash frozen in liquid nitrogen within 2 hr of collection and stored at −20°C.

### Chlorophyll

Chlorophyll *a* concentrations were used as a proxy for phytoplankton biomass. Chlorophyll *a* was measured both *in vivo* and from extracted samples. *In vivo* measurements were made weekly using a hand-held fluorometer (Aquafluor, Turner Designs Inc., USA) for four weeks at 20 stations (Fig. 1) around Santiago Island to track the influence fish pen effluent. This hand-held *in vivo* fluorometer, coupled with a hand-held Global Positioning System (GPS) unit, allowed for real-time data collection regarding the depth and location of these hotspots. Extracted chlorophyll *a* samples were taken from a subset of these sites each week to create a standard curve that allowed the *in vivo* Relative Fluorescence Unit (RFU) readings to be translated into µg Chl *a*/L. Samples for extracted readings were also taken at each transplant sampling time point. For all extracted samples, seawater (50 ml) was filtered onto Whatman GF/F filters (25 mm) and stored in the dark at −20°C until processing. Samples were processed using the method described by Holm-Hansen et al. (1967). Briefly, filters were extracted in 5–10 ml methanol for two hours and fluorescence measured using a Turner Designs 700 fluorometer. Extracts were acidified and remeasured to determine total phaeophytin.

### Dissolved organic carbon

Seawater aliquots (30 ml) were filtered through Whatman GF/F filters (25 mm diameter). The filtrates were acidified and analyzed for total organic carbon (TOC) content (Scripps Institution of Oceanography, Aluwihare Laboratory) on a Shimadzu TOC-V instrument fitted with an autosampler. Briefly, the concentration of each sample was calculated from an average of four 100 µL injections using a five-point potassium hydrogen phthalate standard curve and certified reference materials (courtesy of Dennis Hansell, Rosenstiel School of Marine and Atmospheric Science).

### Bacteria and VLP abundances

Water samples were fixed with a 2% final concentration of 0.02 µm filter sterilized formaldehyde. The “particle” fraction was removed by pre-filtering through a 3 µm polycarbonate filter. Two to three milliliters of filtrate were put on a 0.22 µm polycarbonate filter to collect the “free-living” fraction of the sample. These samples were dried, wrapped in aluminum foil, and stored at −20°C. A quarter of each 0.22 µm filter was prepared for epifluorescence microscopy using Vectashield mounting medium containing DAPI (Vector Laboratories, USA), while the remainder of the filter was archived. Bacteria were counted in 20 haphazardly chosen fields of view (with one 100 µm×100 µm grid per field) at 1000×magnification on an Olympus IX-50 microscope. Ten haphazardly chosen fields were photographed at 1000x on a Nikon, Eclipse TE-2000U using NIS Elements software program to count the frequency of dividing cells (FDC) following the protocol described by Hagstrom et al. [38].

Plate counts were performed to quantify the number of culturable bacteria. Water samples (50–200 µl) from each site were spread onto Thiosulfate Citrate Bile Sucrose (TCBS) agar to select for *Vibrios* and Zobell Marine agar with 25 µg/ml Kanamycin to select for Kanamycin-resistant bacteria. Plates were incubated overnight at 30°C and colonies were counted 12 hr later.

Virus-like particles (VLPs) were quantified on 0.02 Anodisc filters (Whatman, USA) that had 500–1,000 µl of sample filtered through them. Filters were stained with 10x SYBR Gold (Invitrogen, USA) for 15 min, dried, mounted to slides using VectaShield (Vector Laboratories, USA). A Nikon Eclipse TE-2000U and NIS Elements software program were used to image and count 20 haphazardly chosen fields per filter.

*PCR amplification*:

DNA was extracted from water filters, coral, and sediment samples using the UltraClean™ Soil Kit (MoBio).

To amplify community 16S rRNA genes for denaturing gradient gel electrophoresis (DGGE) analyses, the variable V3 region was targeted using primer 341f with a GC clamp (5′-CGCCCGCCGCGCGCGGCGGGCGGGGCG GGGGCACGGGGGGCCTACGGGAGGCAGCAG-3′) and primer 534r (5′-ATTACCGCGGCTGCTGG-3′) [39] following the amplification protocol described by Garren et al. [17]. The PCR products were separated by electrophoresis on a 1.0% agarose gel for confirmation of ∼200 bp product and quantified using PicoGreen (Molecular Probes) following manufacturers instructions using a SpectraMax M2 plate reader (Molecular Devices, USA).

To amplify from all samples for clone libraries of community 16S rDNA genes, a nested PCR was performed using the universal primer 27F (5′-AGAGTTTGATCM TGGCTCAG-3′) and the Eubacterial-specific primer 1492R (5′-TACGGYTACCTT GTTACGACTT-3′; [40]) in a 15 cycle amplification using a 55°C annealing temperature. One microliter of the product was used as the template for the second PCR reaction. Primers 341-forward (without GC clamp [39],) and 981-reverse (41) were used under the same conditions as for DGGE. Three microliters of the final PCR product was used as template for ligation and clone library construction (see below).

To assess the prevalence of this genus in samples that may have had abundances too low to detect via DGGE, we designed specific to probe environmental DNA samples. Genus specific primers were designed using NCBI's Primer-BLAST for a 576 base pair region of *Desulfovibrio spp.* to look for these phylotypes previously associated with Black-Band Disease (BBD) in corals. Primers 645F (CAAGCCCCCAACA CCTAGTA) and 1220R (TACCGTGGCAACGATGAATA) were used in the following PCR protocol: an initial 94°C denaturing step for 5 min was followed by 34 cycles of amplification (45 sec denaturation at 94°C; 45 sec at 53.5°; 2 min extension at 72°C), and a final extension of 10 min at 72°C. Primer specificity was verified via cloning of PCR product.

### Cloning

PCR products from half of the fragments analyzed via DGGE were cloned using Invitrogen's pCR4-TOPO for sequencing kit with Top-10 chemically competent cells following manufacturer's instructions. 48 or 96 colonies were picked for each cloning reaction and transferred into LB + Kanamycin media containing 10% glycerol for a 12 h incubation at 37°C, then submitted to a commercial sequencing service (Agencourt Genomic Services, MA, USA). Briefly, inserts were amplified using the M13 forward primer, sequencing reactions were performed using BigDye Terminator v3.1 (Applied Biosystems), and sequences were delineated using a PRISM™ 3730xl DNA Analyzer (Applied Biosystems).

### Denaturing gradient gel electrophoresis (DGGE) analyses

PCR products (∼200 bp) were separated by GC-content using a hot-bath DGGE system (CBS Scientific). One hundred nanograms of each PCR product were loaded onto 8.0% polyacrylamide gels in 0.5x TAE buffer (20 mM Tris, 10 mM sodium acetate, 0.5 mM Na_2_EDTA, pH 8.2) with top-to-bottom denaturing gradients of 35–65% formamide and urea (100% denaturant being 40% [v/v] formamide and 7M urea). Electrophoresis was run at 55 V for 18 h at 60°C in 0.5x TAE. After electrophoresis, the gels were stained for 15 min in 0.5x SYBR Gold (Invitrogen) in TAE buffer. Gels were then imaged using a UVP Epi-chemi Darkroom with a charge coupled device (CCD) camera.

Community DGGE profiles were compared across sites and sample type using a standard ladder made from 100 ng each of two isolates collected from the fish pen site in January 2007 [17]. Bands of the same relative position among sites or sample type were identified. These select bands were excised from the gel and eluted using the protocol described by Long and Azam [42]. The bands were re-amplified and run on a new gel to confirm the position relative to a known standard. For those products of the correct position, the original excised band was again amplified (without GC-clamp) and the product used for cloning reactions (see Cloning).

### Sequence analyses

Sequence data were trimmed, cleaned, and aligned using Sequencher 4.5. Final clean sequences were exported in FASTA format and imported into the Ribosome Database Project (RDP-10) online portal [43]. RDP was used to align sequences and classify sequences and to compare libraries. Where sequences from the various sample types clustered together, those sequences were hand aligned in Sequencher 4.5 and blasted in NCBI to determine putative identification. Sequences were submitted to GenBank (accession numbers GQ412750 - GQ413933). 99% similarity clusters were used to identify sequences affiliated with a specific organism to eliminate bias induced by Taq polymerase error [44]. Values for the Shannon-Weiner Index of diversity were calculated for clone libraries using FastGroupII program [45].

## Supporting Information

Figure S1 Image showing the transplantation table set-up at the Reference site.
(7.68 MB TIF)
