# Supplementary figures and images for: Resilience of Coral-Associated Bacterial Communities Exposed to Fish Farm Effluent
Source: PLoS One. 2009 Oct 6;4(10):e7319. doi: 10.1371/journal.pone.0007319 (PMC2751826; doi:10.1371/journal.pone.0007319)

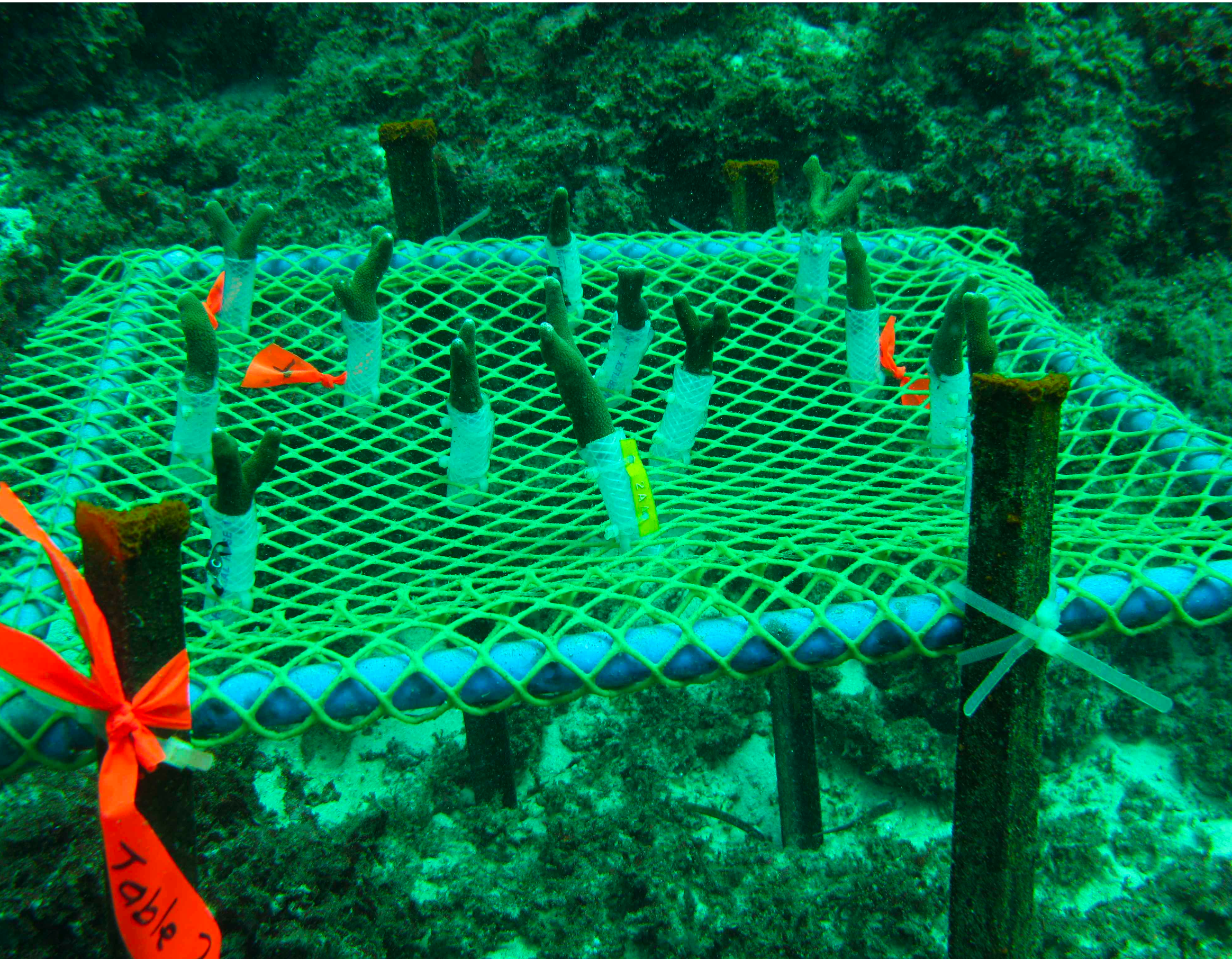

Supplement: Figure S1 — Image showing the transplantation table set-up at the Reference site. (7.68 MB TIF) [file pone.0007319.s001.tif]
